# Unravelling the temporal dynamics of community functions in protists induced by treated wastewater exposure using metatranscriptomics

**DOI:** 10.1038/s41598-025-10083-1

**Published:** 2025-07-04

**Authors:** Manan Shah, Guido Sieber, Aman Deep, Daniela Beisser, Jens Boenigk

**Affiliations:** 1 Biodiversity, University of Duisburg-Essen, Essen, Germany; 2 Present Address: Environmental Metagenomics, Research Center One Health Ruhr of the University Alliance Ruhr, University of Duisburg-Essen, Essen, Germany; 3 Department of Engineering and Natural Sciences, Westphalian University of Applied Sciences, Recklinghausen, Germany; 4 Centre for Water and Environmental Research, University of Duisburg-Essen, Essen, Germany

**Keywords:** Computational biology and bioinformatics, Microbiology, Ecology, Environmental sciences

## Abstract

The discharge of treated wastewater (TWW) into freshwater ecosystems poses a significant impact on microbial communities, particularly protists, which play a crucial role in nutrient cycling and ecosystem stability. While the ecological effects of TWW on microbial diversity have been studied, understanding the functional responses of protist communities remains limited. This study employs metatranscriptomics to unravel the temporal dynamics of protist community functions in response to TWW exposure. Using mesocosm experiment, water samples were analyzed over a ten-day period to monitor shifts in metabolic pathways and community interactions. Our results indicate that processed metatranscriptomic data, focusing on treatment-significant pathways, is more sensitive than traditional methods, such as meta-barcoding, and non-target screening, in detecting wastewater-induced perturbations. Early exposure to TWW significantly altered expression of pathways associated with signal transduction and environmental interaction, while general metabolic pathways showed resilience. Over time, the protist community showed signs of adaptation with expression levels stabilizing towards the end of the experiment. This study underscores the importance of focussing on functional shifts rather than just taxonomic changes for assessing wastewater impacts on freshwater ecosystems. Our findings advocate for the use of metatranscriptomics as a robust indicator for TWW detection, aiding in development of targeted environmental management strategies.

**Supplementary Information:**

The online version contains supplementary material available at 10.1038/s41598-025-10083-1.

## Introduction

Wastewater treatment is a cornerstone of modern environmental management, playing a vital role in mitigating the adverse impacts of urban and industrial wastewater discharge on aquatic ecosystems. Despite significant advancements in treatment technologies, the discharge of treated wastewater (TWW) remains a substantial contributor to environmental pollution, exerting profound effects on microbial community dynamics and ecosystem functioning^1–3^. Among the myriad of microorganisms inhabiting aquatic environments, protists represent a diverse and ecologically significant group, contributing to nutrient cycling, organic matter decomposition, and overall ecosystem stability^4,5^. Understanding the responses of protist communities to TWW exposure is essential for comprehensively assessing the ecological consequences of wastewater discharge and developing targeted management strategies to safeguard aquatic ecosystem health.

Protists exhibit remarkable adaptability and resilience to environmental perturbations, enabling them to persist and thrive in diverse aquatic habitats, including those impacted by wastewater effluents^6,7^. Previous studies have documented shifts in protist community composition following wastewater discharge, characterized by alterations in species abundance and community structure^8,9^. Furthermore, wastewater exposure can induce changes in protist metabolic activities, influencing crucial biogeochemical processes such as carbon and nitrogen cycling, as well as the degradation of organic pollutants^10,11^.

Microbes have been utilized as bioindicators for water quality analysis since the late 19th and early 20th centuries, a practice that has evolved with advancements in microbiology and environmental science. Initially, the detection of pathogens like *Escherichia coli* in freshwater was central to assessing contamination, particularly from human wastewater^12^. The presence of fecal coliforms, including *E. coli*, became a standard measure for evaluating the safety of drinking water and the extent of pollution in natural water bodies. Over time, microbial indicators expanded beyond pathogens to include a broader range of microorganisms, such as *Enterococci* and *Clostridium* species, which signal the presence of untreated or poorly TWW in freshwater systems^13^. Modern techniques, including molecular methods like qPCR and metagenomics, have further refined the detection and quantification of microbial communities, enabling more precise monitoring of water quality and the differentiation between sources of contamination, such as distinguishing between human and animal waste. These advances have been critical in managing freshwater resources, ensuring safe drinking water, and maintaining ecosystem health.

Recently Signal-to-Noise ratio (SNR) has been used in attempt to identify which technique is more effective to identify presence of TWW in a mesocosm study^14^, this study focussed on taxonomic diversity (16 S rRNA and 18 S V9 rRNA) and chemical signals (non-target screening, NTS). SNR can be a critical metric in biological research to quantify the clarity of signals against background noise. In the context of environmental studies, a high SNR indicates a clear distinction between the treatment effects and natural variability, making it a valuable tool for assessing the impact of treatments like wastewater exposure on microbial communities. By analyzing SNR, we can determine the effectiveness of various methods in detecting subtle biological changes induced by environmental stressors.

Building upon this prior research, which compared different types of microbial community data including 18 S V9 rRNA gene sequencing, metagenomics, and non-target data, our study focuses on leveraging targeted metatranscriptomics to elucidate the temporal dynamics of protist metabolic pathways in response to treated wastewater exposure. Metatranscriptomics provides a high-resolution view of gene expression profiles across multiple taxa, offering insights into functional responses that may not be captured by taxonomic analyses alone^15,16^. This approach allows for the simultaneous analysis of active metabolic processes, thereby providing a dynamic picture of microbial activity and adaptability.

In aquatic environments, microbial metabolic pathways and genes related to environmental interactions play crucial roles in determining how communities adapt to changes, such as those induced by the influx of TWW^17^. In natural freshwater systems, pathways involved in general metabolism—such as glycolysis, the tricarboxylic acid (TCA) cycle, and amino acid biosynthesis—are typically dominant, reflecting the stable, nutrient-cycling processes that sustain microbial life^18^. However, when freshwater is exposed to TWW, there is often a marked shift in the microbial community’s functional profile.

Specialized environmental interaction pathways, including signal transduction, cell adhesion, and biofilm formation, become more pronounced in these impacted waters. These pathways are essential for microbial adaptation to environmental stressors, enabling microbes to detect and respond to chemical signals, adhere to surfaces, and form protective communities. This shift suggests that microbes in TWW-affected freshwater are under selective pressure to interact more closely with their environment, possibly due to the presence of contaminants and altered physicochemical conditions. Consequently, genes involved in stress response, detoxification, and resistance to pollutants also become more active in these environments, and for microbial populations these can be heavily aided by secondary metabolites^18,19^.

Understanding these shifts in metabolic and environmental interaction pathways is crucial for predicting the ecological impacts of TWW on freshwater systems, particularly in terms of how microbial communities adapt and potentially influence water quality. For this study, KEGG enrichment analysis was performed for each time point, comparing treatment and control samples, to provide a detailed view of pathway-specific responses over time. KEGG enrichment helps identify significantly overrepresented pathways in response to environmental changes, linking gene expression to functional outcomes. Additionally, clustering of KEGG orthologs and pathways^20^ enables the grouping of genes and metabolic functions with similar expression patterns, facilitating the identification of co-regulated pathways and their temporal dynamics. These tools are essential for understanding how treated wastewater impacts specific metabolic functions and the overall ecological role of protist communities.

Despite advancements in understanding the effects of TWW on microbial communities, significant knowledge gaps persist regarding the differential responses of protist communities at taxonomic and functional levels^21^. Specifically, the resilience of protist functional responses compared to taxonomic changes following wastewater exposure remains poorly understood^22,23^. Additionally, the sensitivity of different metabolic pathways in protists to wastewater-induced perturbations and their subsequent implications for ecosystem functioning warrant further investigation. Bridging these gaps is crucial for advancing our understanding of protist community dynamics in response to wastewater exposure and for developing targeted mitigation strategies to safeguard aquatic ecosystem health.

The present study aims to address these knowledge gaps by undertaking a comprehensive investigation into the differential effects of treated wastewater on protist community functions. Central to this investigation is the elucidation of the temporal dynamics of metabolic pathways in protists following wastewater exposure. Leveraging targeted metatranscriptomics, a powerful approach that allows for the simultaneous analysis of gene expression across the community, we will interrogate changes in protist community structure and gene expression in response to treated wastewater exposure. Bioinformatic tools and statistical analyses will be employed to discern differential responses in metabolic pathways and to assess the relative contributions of taxonomic and functional changes.

This study is guided by two key hypotheses. First, we propose that microeukaryotic metatranscriptomes are a more sensitive method for detecting the presence of treated wastewater in river water compared to other microbial community data types. Second, we hypothesize that changes in protist metabolic pathways not directly tied to growth, such as those involved in secondary metabolism and environmental interactions, will exhibit more pronounced and distinct temporal shifts in response to treated wastewater exposure than general metabolic pathways, like amino acid metabolism, the TCA cycle, and DNA repair. These shifts are expected to reflect the adaptive capacity of protist communities, providing deeper insights into the use of metatranscriptomics as an effective indicator for identifying treated wastewater in freshwater systems.

## Results

### Sequencing information

A total of over 962 million read pairs that passed quality checks were obtained for the 42 samples, the distribution of which is shown in Table 1. These reads were assembled into over 38 million contigs, with an average of 907,092 contigs per sample (standard deviation (σ): 241,553). The highest number of contigs was observed in sample T3_S1, while the lowest number was found in sample C1_S2. On average, 5232 (σ: 471) KEGG orthologs were identified per sample, constituting approximately 24.5% (σ: 5.8%) of the reads that passed quality control. In total, 6,273 unique KEGG orthologs were identified across all reads, which fall into 253 different metabolic pathways.

**Table 1:** Sequencing output and information of quality checked reads, number of assembled contigs, and information on KEGG annotations for the reads and contigs.

Sample Name	Condition	Tank	Time (Hr)	QC Passed Pairs	Assembled Contigs	Reads Assigned to KOs	% KOs Assigned	KOs Identified
C1_S1_R	Control	C1	1	24,224,483	1,021,610	6644711.495	27.42973501	5436
C2_S1_R	Control	C2	1	21,565,157	1,166,264	5403970.238	25.05880313	5499
C3_S1_R	Control	C3	1	19,626,005	850,014	4800620.97	24.46051028	5499
T1_S1_R	Treatment	T1	1	20,950,178	1,342,208	6318233.678	30.15837707	5840
T2_S1_R	Treatment	T2	1	21,954,703	1,351,191	6679742.508	30.42510986	5795
T3_S1_R	Treatment	T3	1	21,842,488	1,582,059	6228768.355	28.51675301	5880
C1_S2_R	Control	C1	12	19,590,330	504,819	4598926.276	23.47549161	3031
C2_S2_R	Control	C2	12	25,066,054	867,259	7331553.553	29.24893385	5443
C3_S2_R	Control	C3	12	24,846,232	712,170	7067504.624	28.44497558	5208
T1_S2_R	Treatment	T1	12	21,825,494	1,226,407	5516279.615	25.2744777	5284
T2_S2_R	Treatment	T2	12	23,536,057	850,620	6909829.167	29.35848246	5143
T3_S2_R	Treatment	T3	12	21,527,768	968,759	5683542.352	26.40098292	5177
C1_S3_R	Control	C1	24	27,914,752	898,792	7251407.703	25.9769734	5415
C2_S3_R	Control	C2	24	22,237,324	865,510	5622427.864	25.28374306	5374
C3_S3_R	Control	C3	24	25,469,235	578,373	7318496.139	28.73465237	5161
T1_S3_R	Treatment	T1	24	26,962,786	585,900	9457243.611	35.07517217	5002
T2_S3_R	Treatment	T2	24	24,872,570	716,731	6954154.842	27.95913266	5009
T3_S3_R	Treatment	T3	24	26,144,361	765,750	8041662.548	30.75868845	5169
C1_S4_R	Control	C1	48	23,956,697	632,963	6211386.251	25.92755692	5171
C2_S4_R	Control	C2	48	25,086,027	820,340	5267487.357	20.99769468	5391
C3_S4_R	Control	C3	48	28,405,356	670,994	6107486.408	21.50117889	5175
T1_S4_R	Treatment	T1	48	20,391,905	899,607	6897296.314	33.82369776	5342
T2_S4_R	Treatment	T2	48	21,438,561	979,473	7028594.86	32.78482572	5478
T3_S4_R	Treatment	T3	48	23,081,006	733,214	5808041.418	25.16372734	5153
C1_S5_R	Control	C1	96	20,733,065	820,740	6886176.014	33.21349744	5523
C2_S5_R	Control	C2	96	21,278,362	920,741	5261405.807	24.72655464	5348
C3_S5_R	Control	C3	96	20,404,896	957,045	5277121.366	25.8620351	5418
T1_S5_R	Treatment	T1	96	23,806,765	1,221,459	6261523.176	26.30144489	5557
T2_S5_R	Treatment	T2	96	23,079,095	1,278,258	5438137.11	23.56304313	5628
T3_S5_R	Treatment	T3	96	21,784,632	1,213,528	5302732.289	24.34161977	5380
C1_S6_R	Control	C1	168	20,664,805	762,577	5165573.814	24.99696375	5303
C2_S6_R	Control	C2	168	21,860,342	985,351	4481173.176	20.49909913	5333
C3_S6_R	Control	C3	168	25,909,121	749,069	5087262.718	19.63502628	5116
T1_S6_R	Treatment	T1	168	29,360,150	649,266	5172056.199	17.61590523	4940
T2_S6_R	Treatment	T2	168	24,231,803	901,232	4754656.072	19.62155301	5294
T3_S6_R	Treatment	T3	168	19,953,383	1,197,516	4267304.255	21.38636969	5495
C1_S7_R	Control	C1	240	21,199,302	617,308	2302062.986	10.8591452	4190
C2_S7_R	Control	C2	240	19,795,995	896,349	2674747.383	13.51155819	4871
C3_S7_R	Control	C3	240	21,412,074	740,929	2103763.858	9.825128841	4487
T1_S7_R	Treatment	T1	240	21,932,510	686,341	3329472.61	15.18053615	4848
T2_S7_R	Treatment	T2	240	21,339,324	1,002,807	3683445.126	17.26130184	5456
T3_S7_R	Treatment	T3	240	20,831,137	906,330	4251955.134	20.41153651	5490

### Metatranscriptomes show added advantage over other methods

**Fig. 1:**
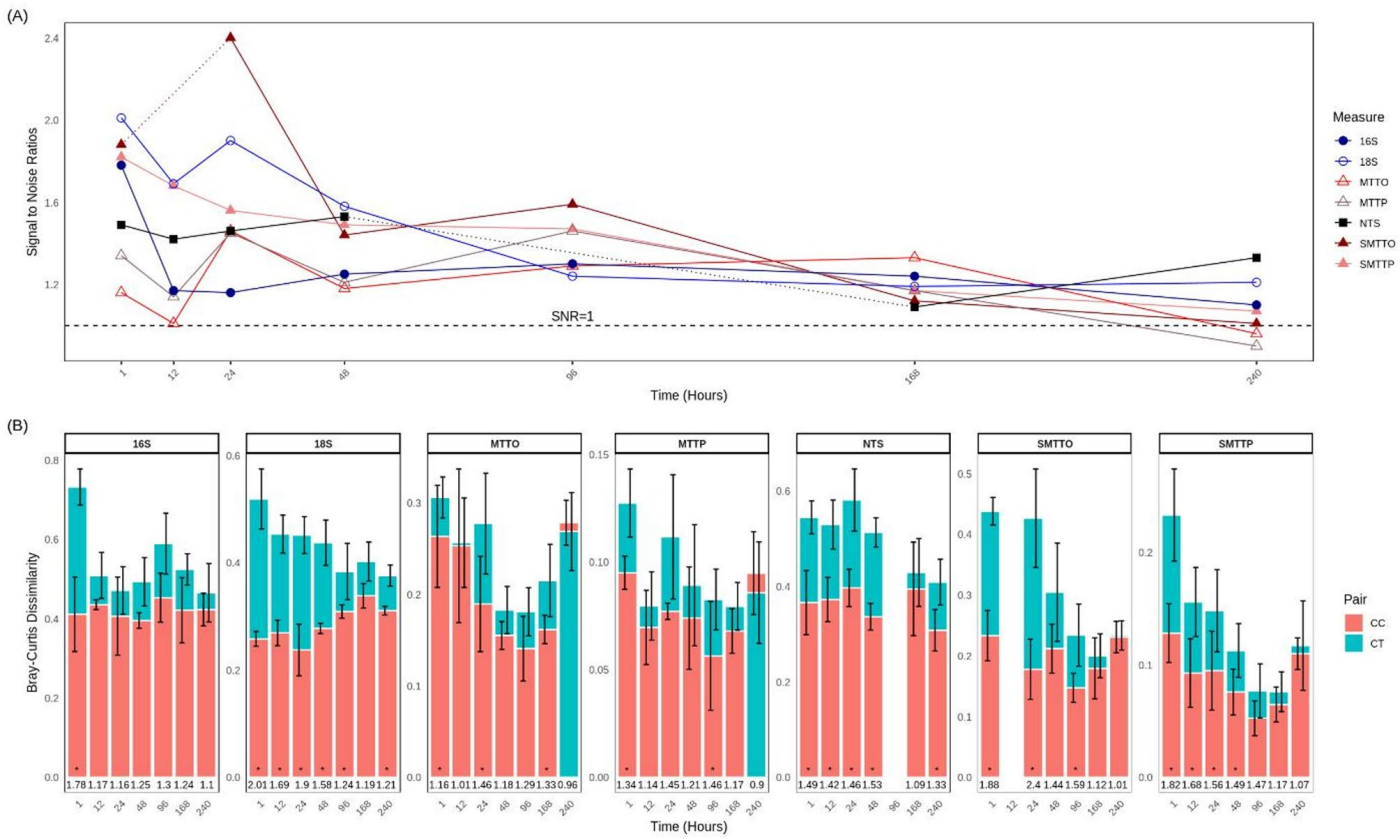
Comparison of various methods for detecting treated wastewater presence in mesocosms. Methods include 16 S rRNA sequencing (for bacterial taxa identification), 18 S V9 rRNA sequencing (for protists), non-target screening (NTS), and two levels of metatranscriptomics: ortholog level (MTTO, SMTTO) and pathway level (MTTP, SMTTP). MTTO and MTTP represent results from complete unfiltered datasets, while SMTTO and SMTTP correspond to datasets filtered using DESeq2 treatment contrast to enhance sensitivity towards treatment effects. The plot illustrates the strength of signals between control-control interactions and control-treatment interactions, represented as signal-to-noise ratios (SNR). Panel (**A**) depicts the comparative trends of SNR values over the experiment duration. Panel (**B**) visually represents the difference between control-control (CC) and control-treatment (CT) interactions, indicating significant differences (p-value < 0.05) with asterisks at the bottom of the bars. Note that at the final time point (240 h), the SNR values for some methods fell below 1, indicating that the signal form control was stronger than from the treatment, as reflected by the reversal of the bar color in panel (B), additionally it should also be noted that 2 values are missing since 2 control replicates had to be discarded.

Immediately after the exposure of treated wastewater (TWW) to the mesocosm system, all methods detected a significant disturbance between the signals from the treatment compared to the control systems. However, this was not consistent across all time points (Fig. 1A, Supplementary Table 1). The signal-to-noise ratios (SNRs) for metatranscriptome samples at the ortholog level (MTTO) and pathway level (MTTP) of metatranscriptomes both showed consistently low SNR values, showing variability at the beginning of the experiment and eventually tapered closer to 1 towards the end of the experiment, falling below 1 at the last time point. This pattern of an initial response, followed by values approaching 1 over time, was similar to those observed with other methods, including non-target-based screening (NTS), 18 S rRNA metabarcoding, and 16 S rRNA metabarcoding. However, these methods maintained an SNR consistently above 1, indicating a stronger signal in the treatment compared to the control (Fig. 1A).

The application of filtering with DESeq2 to represent the treatment effect, as represented by the SMTTO (**s**ignificant **MTT** **o**rthologs) and SMTTP (**s**ignificant **MTT** **p**athways) datasets, resulted in consistently higher SNRs, demonstrating an improved sensitivity in detecting treatment effects throughout the experiment. With SMTTO having the highest SNR value at 24 and 96 h.

Various methods showed the significant SNRs (i.e. the ratio of control-control distances and control-treatment Bray-Curtis distances) at different time points. For instance, 18 S exhibited the best result at 1 h (SNR: 2.01) and 48 h (SNR: 1.58), while SMTTO outperformed other methods at 24 h (SNR: 2.4) and 96 h (SNR: 1.59). At 12 h, both SMTTP and 18 S demonstrated similarly high SNRs. However, at 168 h, only unfiltered MTTO provided significant results, and at 240 h, NTS showed the highest SNR (Fig. 1A).

Additionally, we observed that significant signal-to-noise ratios for metatranscriptomics were often accompanied by relatively lower Bray-Curtis distances (Fig. 1B), especially at the pathway level. This was in stark contrast to methods such as 16 S, which consistently showed high Bray-Curtis distances but fewer time points with significant SNRs (Fig. 1B).

### Metabolism vs. community interaction

Pathways that were enriched in control samples and treatment samples (Table 2) showed that control samples, especially in the initial time points, had higher occurrences of metabolic pathways. On the other hand, the treatment over the course of time showed enrichment of signal transduction pathways and community interaction pathways, with most of them peaking around the 48 h. Following which at 168th and 240th hour marks there were no KEGG orthologs that were significantly enriched in treatment or control.

**Table 2:** Pathway level classification of the KEGG orthologs enriched either in treatment or control at different sampling timepoints (in Hours) obtained after KEGG enrichment analysis. The “*” denotes the time points where the pathways were significantly enriched in treatment/control conditions.

				Control							Treatment						
Pathway_ID	Cluster Number	Subsystem	Pathway Names	1 H	12 H	24 H	48 H	96 H	168 H	240 H	1 H	12 H	24 H	48 H	96 H	168 H	240 H
map04218	0	Cellular Processes; Cell growth and death	Cellular senescence												*		
map04810	1	Cellular Processes; Cell motility	Regulation of actin cytoskeleton											*			
map04550	1	Cellular Processes; Cellular community - eukaryotes	Signaling pathways regulating pluripotency of stem cells											*			
map04010	1	Environmental Information Processing; Signal transduction	MAPK signaling pathway									*	*	*			
map04013	1	Environmental Information Processing; Signal transduction	MAPK signaling pathway - fly											*			
map04015	1	Environmental Information Processing; Signal transduction	Rap1 signaling pathway											*			
map04068	1	Environmental Information Processing; Signal transduction	FoxO signaling pathway											*	*		
map04071	1	Environmental Information Processing; Signal transduction	Sphingolipid signaling pathway									*		*	*		
map04072	1	Environmental Information Processing; Signal transduction	Phospholipase D signaling pathway											*			
map04370	1	Environmental Information Processing; Signal transduction	VEGF signaling pathway											*			
map04668	1	Environmental Information Processing; Signal transduction	TNF signaling pathway									*	*	*			
map00561	0	Metabolism; Lipid metabolism	Glycerolipid metabolism	*													
map00230	0	Metabolism; Nucleotide metabolism	Purine metabolism				*										
map01100	0		Metabolic pathways	*	*	*	*										
map01110	0		Biosynthesis of secondary metabolites	*	*	*	*										
map01240	0		Biosynthesis of cofactors		*	*											
map01250	0		Biosynthesis of nucleotide sugars	*	*												

### Variation of ortholog expressions over time

**Fig. 2:**
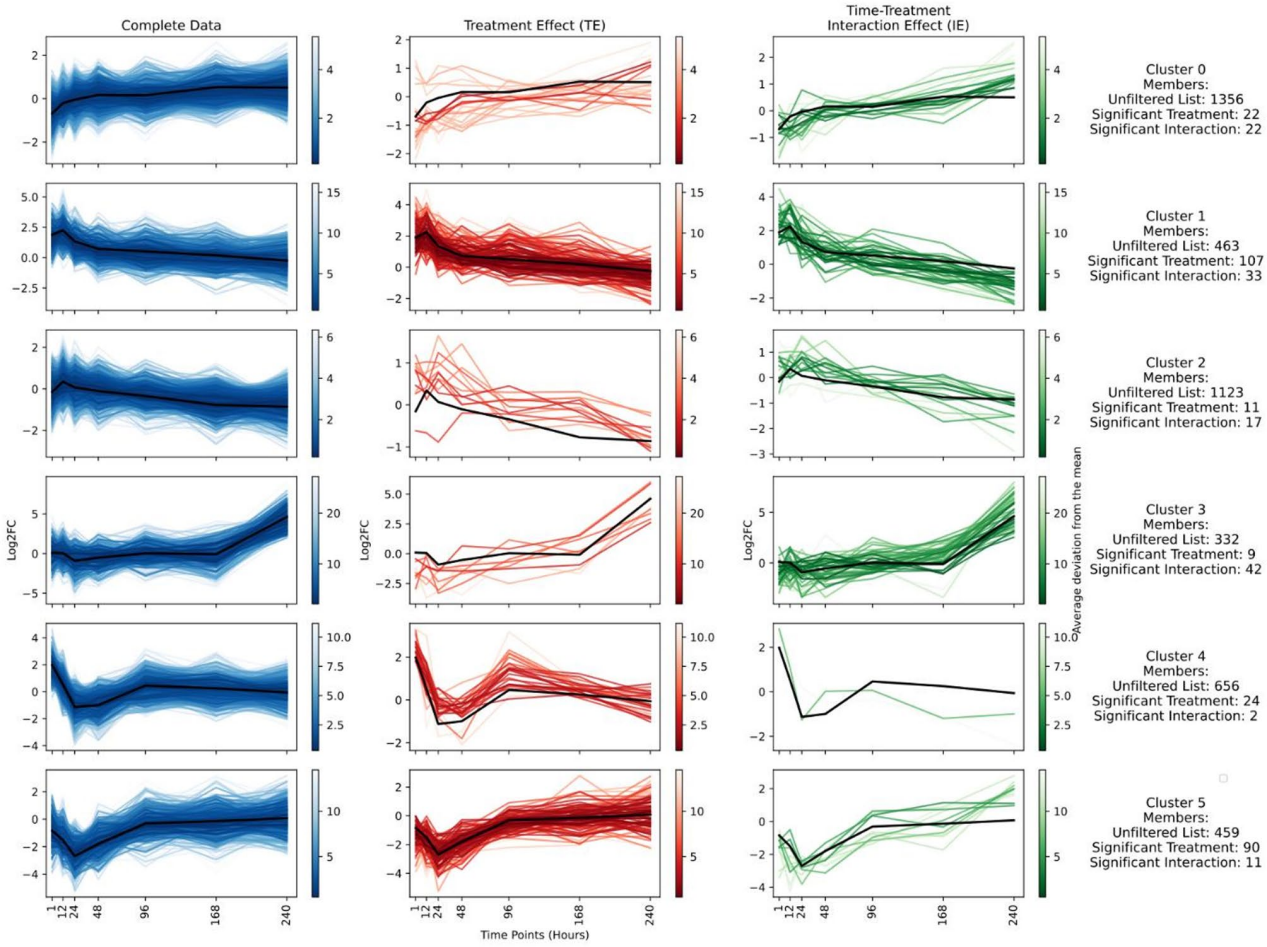
KEGG ortholog clustering. Variation in log2 fold change over time for all KEGG orthologs (first column on the left), KEGG orthologs from this cluster identified as significant by DESeq2 with treatment as the contrast (middle column, TE), and orthologs from this cluster identified as significant while considering the interaction of treatment and time as contrast in DESeq2 (right column, IE). The black line in each plot represents the median line from the unfiltered dataset. Colour gradation in all three columns indicates the deviation of the orthologs from this median line.

Out of the total 6,273 KEGG orthologs identified in all samples combined, 330 were significantly differentially abundant between treatment and control samples [showing treatment effect (TE)], and 177 were significantly differentially expressed when considering the interaction with time [showing interaction effect(IE)]. Clustering of KEGG orthologs into six distinct groups revealed the temporal dynamics of gene expression profiles in response to wastewater treatment (Fig. 2). Each cluster exhibited unique patterns, with significant treatment-specific responses particularly evident in the early hours post-treatment.

The orthologs in cluster 0 were under-expressed immediately after the exposure to TWW, but their expression levels recovered and stabilized close to zero (no effect) towards the end of the experiment. Within cluster 0, the TS orthologs showed two distinct patterns: some orthologs had an initial log2 fold change close to −1 (more expressed in control samples), while others had an initial log2 fold change close to + 1 (more expressed in treatment samples), both converging towards zero over time. The orthologs starting at + 1 were no longer seen in the IE clusters, highlighting a unique split in cluster 0.

Cluster 1 started with a relatively high log2 fold change of around + 2, indicating approximately four times higher expression in treatment samples initially. This expression peaked at 12 h before returning to near baseline levels over the 10-day period. In contrast, orthologs in clusters 2 and 3 initially showed similar expression levels in control and treatment samples (log2 fold change close to 0). Over time, cluster 2 orthologs became more abundant in control samples, while cluster 3 orthologs were more abundant in treatment samples.

Orthologs in cluster 4 exhibited a different pattern, with high expression in treatment samples at 1 and 12 h (log2 fold change around + 1). Their expression levels then dropped to less than one-fourth of the control values around 24 h, before becoming similar in both control and treatment samples over time. Conversely, orthologs in cluster 5 started with similar expression levels in control and treatment samples at 1 h but showed a sharp decrease in treatment samples around 24 h, eventually recovering to control levels.

### Functional changes inferred from changing pathways over time

**Fig. 3:**
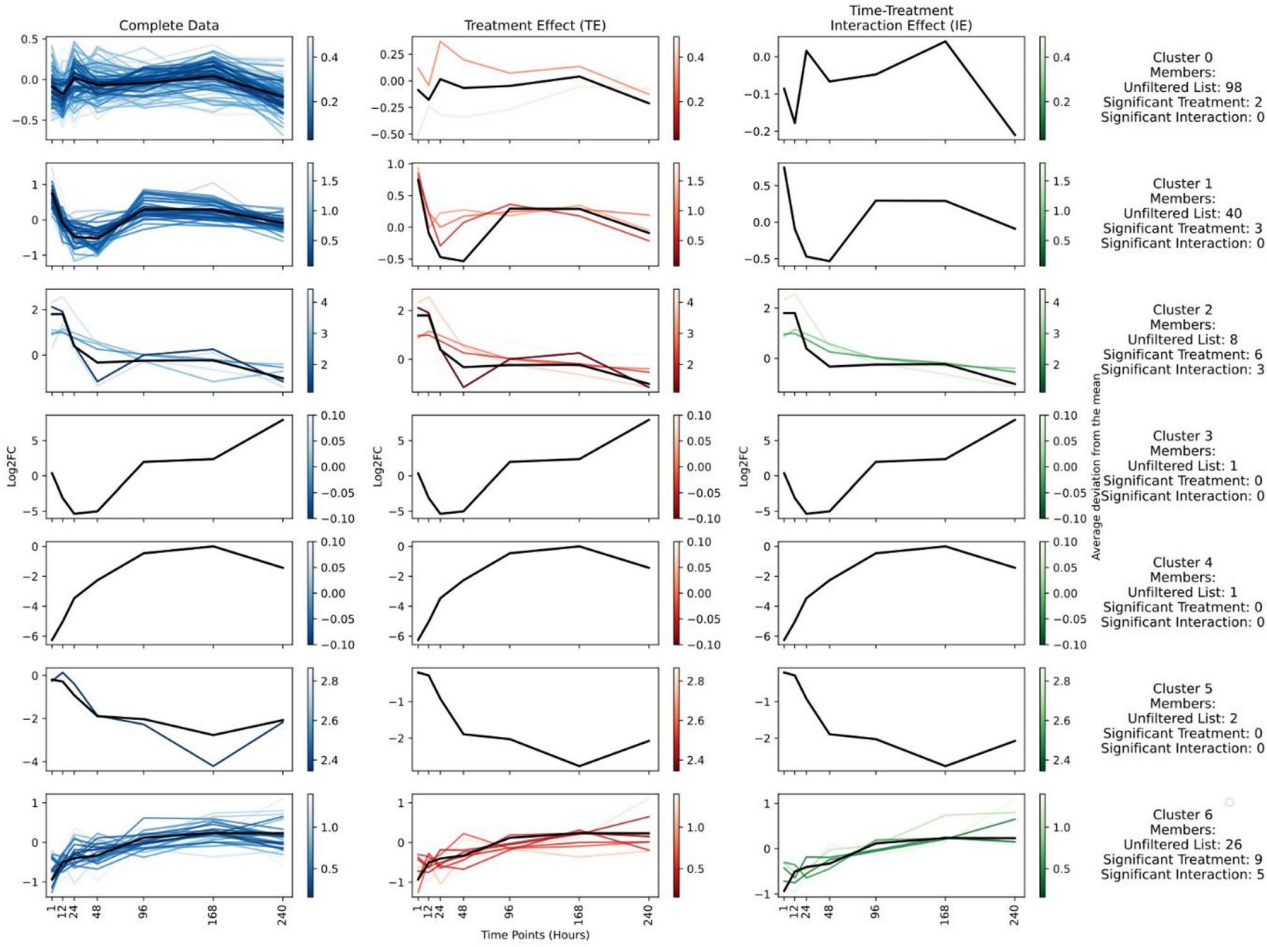
KEGG pathway level clustering. Variation in log2 fold change over time at pathway level for all KEGG orthologs (first column on the left), KEGG pathways from this cluster identified as significant by DESeq2 with treatment as the contrast (middle column; TS), and pathways from this cluster identified as significant while considering the interaction of treatment and time as contrast in DESeq2 (right column; IS). The black line in each plot represents the median line from the unfiltered dataset. Colour gradation in all three columns indicates the deviation of the orthologs from this median line.

At the pathway level, clustering provided broader insights into the community functions of protists to TWW exposure (Fig. 3, Supplementary Table 2). The three clusters with the most pathways assigned to them were clusters 0, 1, and 6, each displaying distinct patterns.

Cluster 0 pathways fluctuated initially but consistently stayed between log2 fold changes of −0.5 and 0.5, indicating similar expression levels in control and treatment throughout the experiment (Fig. 3). However, only two pathways related to energy metabolism and DNA repair were identified as significant in treatment effect clusters (TE), and none in the interaction with time (IE) clusters (Supplementary Table 2).

Cluster 1 pathways began with a log2 fold change of + 1, indicating double the expression in treatment compared to control. Over the first two days, this expression fell below zero, meaning their expression dropped below control levels, and eventually stabilized close to zero for the remainder of the experiment (Fig. 3). The TS pathways in this cluster included those involved in signal transduction, apoptosis, and isoquinoline alkaloid biosynthesis. Similar to Cluster 0, no pathways in Cluster 1 were seen as significant in the IE clusters (Supplementary Table 2).

Cluster 6 contained 26 pathways, which initially had half the expression level in treatment compared to control. By the fourth day, the expression levels in treatment and control had become similar and remained stable for the rest of the experiment (Fig. 3). This cluster had 9 pathways in the TE list and 5 in the IE list. The TE pathways were predominantly involved in basic metabolism, including amino acid metabolism, starch and sucrose metabolism, and flavonoid biosynthesis. Other pathways involved in environmental interactions, such as signal transduction and RNA polymerase synthesis, were also present. Notably, some signal transduction pathways, flavonoid degradation, and beta-alanine metabolism pathways were included in the IE pathways, along with an additional metabolic pathway for glycan biosynthesis, which was absent from the TE significant pathways (Supplementary Table 2).

Further analysis of pathway clusters revealed distinct associations with protist taxa, though these associations were not exclusive. Clusters supported by many pathways, particularly Clusters 0, 1, and 6, correlated with only a few taxa, which were distributed across diverse taxonomic groups. In contrast, clusters supported by fewer pathways, such as Cluster 2, were linked to more taxa and showed stronger associations with distinct taxonomic groups, including OTUs from Amoebozoa (Supplementary Fig. 1, Supplementary Table 3).

## Discussion

The detection of treated wastewater (TWW) in freshwater ecosystems is of paramount importance due to its significant ecological, public health, and regulatory implications^24,25^. Despite undergoing advanced purification processes, TWW may still harbor residual contaminants, including pathogens, pharmaceuticals, personal care products, industrial chemicals, and nutrient loads such as nitrogen and phosphorus^26,27^. The current study aimed to investigate the temporal dynamics of protist responses to TWW using metatranscriptomics, focusing on the sensitivity of different microbial community data types. While several popular and relatively straightforward methods, such as metabarcoding for biological community analysis and non-target screening for chemical analysis, are available for detecting the presence of TWW, they do not always provide comprehensive information^28^.

In our study, we expanded upon the experimental framework of Sieber et al. (2023), incorporating metatranscriptomic data to analyse both changes on gene and pathway level using the KEGG database. We found that although metatranscriptomics data were not the most sensitive for detecting TWW exposure, particularly at the ortholog level, they were exceptionally informative at the pathway level for identifying functional changes over time. Metatranscriptomics (MTT) has demonstrated its value in wastewater treatment systems to assess treatment efficacy and identify functional attributes such as antibiotic resistance^29,30^. However, its application in environmental samples to trace TWW remains underexplored. One possible reason for this gap is the limited availability of curated metatranscriptomic resources, with only 0.2% of UniProtKB entries being reviewed and verified (as per their website in September 2024). In contrast, the KEGG database offers over 27,000 orthologs from more than 550 pathways. The conserved nature of orthologous genes across species facilitates functional identification, suggesting that even a relatively small knowledge base can be effectively leveraged for metatranscriptomic analysis. Therefore, despite metatranscriptomics not being the most sensitive indicator of TWW, it provides a detailed understanding of how microbial communities react to TWW-induced disturbances at a functional level, particularly when focussing on pathway-level changes.

### Despite the potential strength of metatranscriptomics a variety of approaches and techniques can be used for water quality assessment

Metabarcoding approaches, like 16 S and 18 S rRNA sequencing, offer a rapid high-resolution taxonomic profiling of microorganisms, providing insights into the diversity and abundance of species present in wastewater effluents^31,32^. Metabarcoding is widely used in environmental monitoring because it is powerful for identifying taxonomic composition, which is essential when tracking changes in response to environmental conditions. Through amplicon sequencing, researchers can detect microbial indicators of water quality and assess the potential impacts of TWW on freshwater ecosystems, including, for instance, the spread of antibiotic-resistant bacteria and pathogenic organisms^33^. One main advantage of amplicon data is the broad background data basis allowing for substantiated comparisons between sampling sites and affiliating an increasing share of sequences with taxonomic information. On the other hand, uncertain taxon assignments in databases, copy-number variations and uncertainties and limitations in linking operational taxonomic units (OTUs) or amplicon sequence variants (ASVs) with distinct species limit the resolution of these proxies. Further, these studies do not usually target functional genes which may be more informative regarding shifts in ecosystem function or the type of pollution, i.e. the chemical substances^34^.

Non-target chemical screening may circumvent some limitations of barcoding approaches and has been used in broad detection of various chemical contaminants, including pharmaceuticals, personal care products, and industrial chemicals that may persist in TWW^35,36^. It provides the chemical “fingerprint” of water samples and therefore directly allows for testing the efficacy of wastewater treatment processes ​^37^. The major advantage of this method lies in its capacity to identify changes in unknown or unexpected compounds. However, it is cost and resource-intensive and requires complex data interpretation, which can be a significant limitation over techniques like metabarcoding^38^. Despite these drawbacks, non-targeted screening remains a robust tool for detecting chemical pollutants, particularly unknown contaminants in aquatic environments^39,40^.​.

Other methods employed for monitoring TWW include metagenomics and quantitative PCR (qPCR)^41^. These methods again focus more on the biological proxies, similar to amplicon data. But metagenomics provides a more comprehensive view of microbial communities by analysing the entirety of the genetic material present in a sample. This approach offers insights into both known and novel microorganisms, as well as their functional potential^42,43^. However, it does not reveal which genes are actively expressed and thus integrates information from active, inactive and dead biological materials^44^. Conversely, qPCR is a targeted method that detects specific pathogens or genes of interest with high sensitivity and specificity, allowing for precise quantification^45^. These methods have been validated across various studies, demonstrating their effectiveness in monitoring the quality of TWW and assessing the environmental impact of wastewater discharge^41^.

The comparative strength of metatranscriptomics in monitoring functional changes induced by TWW aligns with our hypothesis and supports the notion that metatranscriptomic approaches provide a high-resolution view of microbial responses to environmental perturbations. The 18SV9 rRNA dataset, while still effective, did not exhibit the same degree of response, underscoring the advantages of using metatranscriptomic data for detecting functional changes within microbial communities. The analysis of pathway clusters highlights metatranscriptomics’ ability to capture functional responses in specific protist communities. In Clusters 0, 1, and 6, while no clear functional patterns were identified, many of the contributing taxa are substrate-bound heterotrophs, emphasizing their potential roles in processing organic matter and maintaining community metabolism under treated wastewater exposure. This suggests that the functional responses in these clusters are likely shaped by broad ecological functions rather than specific stress-related pathways.

However, assigning a unidirectional causal relationship—whether taxonomy drives functional patterns or vice versa—is speculative. Instead, the observed patterns likely reflect a dynamic interplay between taxonomy and function shaped by environmental pressures. Metatranscriptomics excels in capturing this interplay, providing a detailed view of microbial community responses to stressors like treated wastewater.

The NTS displayed variable sensitivity over time, and while it was effective at certain time points, it did not consistently outperform the metatranscriptomic pathway-level (MTTP/SMTTP) and ortholog-level (MTTO/SMTTO) analyses, reinforcing the complementary role of metatranscriptomics in environmental monitoring. There are some recent studies that support the superiority of metatranscriptomics in environmental disturbance monitoring. For instance, the chapter by Gonçalves et al.(2023)^46^, shows that metatranscriptomics can show more subtle changes in functions than just the metabarcoding or metagenomics techniques. Additionally, it is also shown that metatranscriptomics is superior and more sensitive since it shows the active transcripts in an environment over the “potential” function that just DNA techniques provide information on^47,48^. Previous research has already established that microbes react to every little change in the environment^14,49–51^, which in turn cause chemical changes which are more relevant to study or identify the potential of indicators of water quality^52^, rather than just using the chemical elements. These results highlight the need for high-resolution methods to capture the complex and rapid changes in microbial communities like metatranscriptomics, which traditional methods might miss.

Lower Bray-Curtis distances observed in metatranscriptomics likely indicate that in general functional stability of the ecosystem is maintained even under perturbation. This aligns with the concept of ecological resilience, where microbial communities maintain key functions despite underlying taxonomic changes^53,54^. Metatranscriptomics captures functional similarity across samples, as transcriptomes are influenced by immediate environmental conditions and functional demands^44^. Even in treatment samples, fundamental metabolic processes and stress responses may converge on similar pathways, thereby reducing overall dissimilarity between control and treatment. This characteristic, combined with relatively high SNRs at specific time points, suggests that while the total variability in transcriptomic profiles is lower, the differences between control and treatment are nonetheless consistent and measurable relative to the inherent noise in the data. In contrast, higher Bray-Curtis distances in metabarcoding or metagenomics data may reflect greater taxonomic variability, which can dilute the control-treatment signal and reduce SNRs at certain times. Additionally, SNRs approaching 1 at the final time points across all methods indicate that inherent differences between control and treatment flumes were minimal by the 10th day, likely reflecting stabilization and adaptation of the microbial communities to the treated wastewater exposure^19^.

The temporal dynamics observed in the SNR analysis and clustering results revealed distinct phases in the microbial community’s response to treated wastewater exposures. Early significant responses, particularly within the first 24 to 48 h, were followed by a period of stabilization. This pattern indicates an initial acute stress response phase, characterized by rapid changes in gene expression, 18SV9 diversity and chemical signals, and a subsequent adaptation phase where the community begins to stabilize and potentially acclimate to the new environmental conditions. These findings were also observed in ways by other researchers that saw eventual recovery of natural conditions after the exposure to such effluents^14,55^, these also show that protist communities exhibit distinct temporal shifts in response to wastewater-induced perturbations, reflecting their adaptive capacity.

### Making the most out of metatranscriptome data as an indicator

The use of metatranscriptomic data for detecting TWW in freshwater, required significant method optimization. This was expected as optimization is crucial when developing methods for indication^32,38^. Although the signal-to-noise ratio (SNR) showed promising results for detecting TWW, the inclusion of both filtered and unfiltered datasets helped reveal the difference between treatment and control (Fig. 1). We applied DESeq2 based filtering to remove orthologs and pathways that did not change significantly between control and treatment, which greatly improved sensitivity at the ortholog level. However, the pathway-level (MTTP and SMTTP) classification consistently provided more informative insights into evolving functions and metabolic activities over time^56,57^. This is evident in our study, where ortholog clustering lacked functional insights, despite distinct expression patterns (Sect. 3.4 and 3.5).

Accordingly, clustering analysis revealed broader and more informative insights into functional responses on the pathway level. For instance, pathways in Cluster 0, which included general metabolic functions like carbohydrate and lipid metabolism (Supplementary Table 2), remained stable throughout the experiment. Such resilient pathways are largely uninformative for TWW detection, since they do not react as strongly to such perturbations. In contrast, specialized pathways, such as signal transduction and xenobiotic metabolism (cluster 1 and cluster 6) displayed a stress response followed by recovery presumably due to acclimatisation. Pathways in Cluster 1 showed an early increase in expression followed by a decline while pathways in cluster 6 showed the opposite pattern, i.e., a lower initial expression. The early increase and subsequent decline in Cluster 1 pathways likely reflects acute stress responses, such as signal transduction and apoptosis, enabling rapid adaptation to environmental changes^58^. Conversely, the delayed yet sustained increase in Cluster 6 pathways, including secondary metabolism and RNA polymerase synthesis, indicates longer-term stabilization and functional recovery^59,60^. These dynamics highlight the interplay between immediate stress responses and gradual functional recovery, reflecting the adaptive capacity of community functions. Additionally, our analysis confirmed that pathways involved in signal transduction, membrane transport, and secondary-metabolite metabolism exhibited greater changes in treatment samples, supporting our hypothesis that specialized pathways are more sensitive to environmental stress. Cluster 6 also included one essential pathway RNA polymerase, which is required by cells to transcribe DNA into RNA. This pathway was presumably upregulated in order to achieve the general stress response mechanisms reflected by the other pathways^61^. Our results are in accordance with findings that general metabolic pathways are resilient to stress^48,62,63^, while specialized pathways, often regulated by signal transduction mechanisms^64^, respond more dynamically^65,66^. The enrichment of these pathways underscores the importance of focusing on specialized metabolic activities to understand the full impact of TWW on microbial communities.

Our findings align with previous studies that documented shifts in microbial community composition and metabolic activities following wastewater discharge^8,9^. The latter study highlights how TWW in irrigation, even with plastic mulch barriers, can alter microbial communities in soil and crops. Similarly, Cuprys et al. (2023) observed that despite functional changes, the overall microbial structure often remains stable, a theme we noted during the stabilization phase after the initial stress response in our study (see also Sieber et al. 2023). This suggests that while meta-transcriptomic data reveal subtle functional shifts, microbial communities may exhibit resilience, complicating the interpretation of long-term ecological impacts.

While this study demonstrates the strengths of metatranscriptomics in water quality assessment, there are limitations that should be acknowledged. The experimental setup, while controlled, may not fully replicate natural environmental conditions, and factors such as environmental variability and the complexity of natural microbial communities could influence the results. Additionally, the focus on a single type of wastewater and a specific geographic location may limit the generalizability of the findings. These challenges underscore the importance to complement metatranscriptomic data with other methods in integrated analyses, across different environmental settings to obtain a more comprehensive understanding.

## Conclusions

This study highlights the varying sensitivity of metatranscriptomic and other approaches in detecting treated wastewater and elucidates the differential and temporal responses of protist communities, while comparing and appreciating the effectiveness of all techniques. By focusing on pathway-level dynamics, our findings emphasize the value of specialized metabolic pathways, such as stress response and signal transduction pathways, in understanding the ecological impacts of wastewater discharge. We demonstrate that metatranscriptomics is a valuable tool for developing targeted environmental management strategies, as its filtered data maintained high sensitivity and discriminatory power throughout the experiment. The enhanced sensitivity and functional insights provided by this approach, especially regarding the pathways identified in clusters with the most pronounced variation, can significantly improve water quality monitoring and guide targeted management strategies to mitigate the ecological impacts of urban and industrial wastewater discharge. However, it is important to note that this work represents a case study conducted under controlled experimental conditions. To translate these findings into real-world management strategies, further investigations in natural environments are required to validate the observed dynamics and assess their broader ecological implications. Such studies will be critical for refining metatranscriptomic approaches and developing targeted interventions to safeguard freshwater ecosystems.

## Methods

### Experimental setup

The experiments were conducted using Aquaflow experimental mesocosms, expansive circular flow systems situated within the biodiversity greenhouses of the University of Duisburg-Essen^67^. Six such systems were employed following the methodology outlined in the paper by Sieber et al.^14^. Briefly, each system comprised three tanks, including one 270-liter (L) tank and two 40 L tanks, interconnected by two sediment-filled channels. These channels, measuring 2 m (m) and 4 m in length respectively, had a width of 10 centimeters (cm) and a depth of 20 cm. An electric pump facilitated water circulation from the last tank to the first. The design and setup of the Aquaflow systems can be found in Stach et al. for further clarification^68^.

For each individual system, 365 L of water (prefiltered with 200 μm nets) and 60 L (approximately 150 kg) of sediment sourced from the Boye River (Bottrop, Germany; coordinates: 51°33’19.7"N 6°56’38.3"E) were collected and utilized. The sediment underwent homogenization before being evenly distributed among the six channels. Similarly, water was collected and acclimatized for 14 days, prior to the commencement of the experiment.

The treated wastewater used in the experiments was obtained from the Emschergenossenschaft’s wastewater treatment plant in Schwerte (Germany). Three of the six systems were kept as control systems (C1, C2, C3) and in the other three systems 33% of the river water was replaced by this treated wastewater (T1, T2, T3). Upon the addition of treated wastewater to the experimental systems, water samples were collected at various time intervals, including 1, 12, 24, 48, 96, 168, and 240 h. These samples were filtered through 0.2 micrometer (µm) filters, air-dried, and stored at −80 °C in a preserving solution(Zymo DNA/RNA shield).

### Nucleic acid extraction

RNA was extracted from the samples using the Zymo Quick DNA/RNA microprep plus kit (Zymo Research Europe, Freiburg, Germany) with a slightly modified protocol. Briefly, two filters of each sample along with the DNA/RNA shield were transferred to bead bashing tubes (Zymo BashingBeads Lysis Tubes (0.1 + 0.5 mm)) and bashed using FastPrep homogeniser (MP Biomedical) for 5 runs of 30 s (s) each at 5.5 m/s, with resting on ice for 1 min between each run. These tubes were then centrifuged for 2 min, after which the supernatant was transferred to fresh tubes and an equal amount of lysis buffer was added. Subsequently, this solution was added to the DNA spin column, and centrifuged, the resultant flow through of this centrifuge was used for RNA extraction. To this flow through 1:1 ration of ethanol was added, and then the resulting solution was added to the RNA spin column and centrifuged to obtain the RNA bound to the column. DNAse I treatment was carried out by first washing the column with wash buffer and centrifugation, followed by treatment with DNAse for 30 min (5 µl of DNAse I + 35 µl water). This column was subjected to multiple rounds of washing with DNA/RNA prep buffer and washing buffer with respective centrifugation, before finally using RNAse free water to extract the purified RNA from the column. For each step the centrifugation was done at room temperature for 30 s at 10,000 rpm, except the last washing step which was centrifuged for 2 min at the same speed.

This RNA was then stored at −80 °C till it was sent for sequencing to Novogene Europe (Cambridge, United Kingdom). The sequencing was done on Illumina NovaSeq6000 on PolyA selected RNA material to obtain only Eukaryotic metatranscriptome sequences, resulting in 150 base-pairs (bp) long, paired-end reads.

### Bioinformatic analysis

The demultiplexed paired-end raw sequences, delivered by the sequencing company, were further processed using the modified Taxmapper supplement pipeline^69^. Briefly, the steps of the modified pipeline are as follows: First is the verification of quality of all the samples using FastQC [v 0.11.9, default parameters^70^,, and MultiQC [v1.8, default parameters^71^,, and trimming of the low-quality reads using cutadapt [v2.9, -q 20 -m 50^72^,. Second is assembly of reads into contigs using rna-spades [v3.15.5, default parameters^73^,, cleaned using AssemblyPostProcessor from PlantTribes2 [v2, prediction type="transdecoder”^74^, and quantification of reads recruited in each contig using Salmon [v1.10.2, default parameters^75^,. Next, functional annotations were obtained against Uniprot and KEGG using rapsearch [v2.24, -b 1 -v 0 -p T -t n -e 1^76^, and kofam_scan [-E 0.05^77^, respectively, and taxonomic annotation using TaxMapper [v1.0.2, default parameters^69^,. From the resultant functional profiles at the ortholog (MTTO) and pathway (MTTP) levels, only reads assigned to a pre-filtered KEGG database were retained. This database was manually curated to include reference data only from protistan organisms, ensuring the removal of fungal and other non-protist eukaryotic entries. While functional overlap across eukaryotic groups may introduce some unavoidable similarities, this curation process significantly minimizes non-protist contributions.

### Signal-to-noise ratio analysis

We processed the sequences following the established standard methods for metatranscriptomes. Specifically, we normalized the final KEGG annotation profiles generated in the previous step using DESeq2 and analysed the different effects [v1.39.8, with design: design = ~ treatment + time + treatment: time, all other parameters were set to their default values, including the thresholds^78^. Scripts for SNR analysis were adapted from Sieber et al. (2023), and applied to obtain signal-to-noise ratios. Likewise, in the Sieber et al. study, all data were processed according to the standard procedures for the respective data types (16 S amplicons, 18SV9 amplicons, and chemical non-target data (NTS)). Dissimilarity was assessed using Bray-Curtis dissimilarity after removing features that were found in only 1 sample, and pairwise Adonis analysis with Benjamini-Hochberg (BH) correction for multiple testing was applied to validate environmental perturbations, the same correction for multiple testing is applied in DESeq2 by default. The Signal-to-Noise Ratio (SNR) was calculated by comparing treatment-control dissimilarities to control-control dissimilarities, and statistical tests (t-tests and Wilcoxon) were used to analyze differences in SNR. Additionally, to focus on the treatment effect, we created an additional filtered datasets. This was achieved by retaining only those orthologs (SMTTO) and pathways (SMTTP) that had an BH adjusted p-values below 0.05 for the treatment contrast from the DESeq2 output. The comparative values for the other methods (16 S, 18 S, NTS) were directly taken from the aforementioned publication. The datasets SMTTO (12 h) had to be excluded, since one replicate in the control sample was an outlier (supplementary Fig. 2), similar to exclusion of NTS (96 h) dataset in Sieber et al.

### Clustering of orthologs and pathways

Log2 fold changes were calculated for each pathway and ortholog over the sampling time-points individually, which was followed by clustering into 5 clusters and 6 clusters, for orthologs and pathways respectively. Clustering was done using the sklearn library [v1.4.2^79^,. For each identified cluster the median line was identified, followed by calculation of deviations from this for each pathway and ortholog by summing the squared errors (SSE) at each time point. These SSE were used to colour the lines and identify the pathways that were closest to the cluster means. The DESeq2 treatment (TS) and treatment + time interaction (IS) contrasts were used to identify significantly different pathways and orthologs between control and treatment (BH adjusted p-value < 0.05) within the clusters.

Additionally, to identify taxonomic groups directly influencing the clusters, we carried out differential abundance analysis for the 18 S dataset at OTU level and correlated (Pearson correlation^80^), the log2 fold changes over time with the cluster means. OTUs positively correlated to any of the clusters with p-values below 0.05 were selected and plotted in a heatmap (complex heatmap package, without clustering the rows and columns^81^),.

### KEGG enrichment analysis

KEGG enrichment analysis was conducted as described in previous work^82^. However, for this study, the analysis was performed for each time point and compared between treatment and control samples. Specifically, significant KEGG orthologs (KOs) identified in the metatranscriptomic data were analyzed using the R package GAGE [ver. 2.44.0^83^, and a hypergeometric test in R. The hypergeometric test is particularly useful in gene set enrichment analysis to determine if there is a non-random association between two categorical variables, aiding in the identification of significantly enriched biological pathways. Significant KOs with a fold-change of at least two and a relative occurrence of at least 10% in either group were used for enrichment analysis. The sets of KOs that were more frequent in either treatment or control samples and present in both were used as test sets, with all identified KOs across all time points as the background set. Pathways were considered significantly enriched with a p-value below 0.01.

## Electronic supplementary material

Below is the link to the electronic supplementary material.

Supplementary Material 1

## Data Availability

All raw meta-transcriptomics data has been made available via NCBI BioProject PRJNA1019091 (Under RNA), all supporting scripts are made available via github https://github.com/mbshah/GW21-MTTs.
